# Development of a radiosensitivity gene signature for patients with soft tissue sarcoma

**DOI:** 10.18632/oncotarget.16194

**Published:** 2017-03-15

**Authors:** Zaixiang Tang, Qinghua Zeng, Yan Li, Xinyan Zhang, Jinlu Ma, Mark J. Suto, Bo Xu, Nengjun Yi

**Affiliations:** ^1^ Department of Biostatistics, School of Public Health, Medical College of Soochow University, Suzhou 215123, China; ^2^ Jiangsu Key Laboratory of Preventive and Translational Medicine for Geriatric Diseases, Medical College of Soochow University, Suzhou, 215123, China; ^3^ Center for Genetic Epidemiology and Genomics, Medical College of Soochow University, Suzhou, 215123, China; ^4^ Department of Biostatistics, University of Alabama at Birmingham, Birmingham, AL 35294, USA; ^5^ Drug Discovery Division, Southern Research Institute, Birmingham, AL 35294, USA; ^6^ Department of Radiation Oncology, The First Hospital, Xi'an Jiaotong University, Xi'an, Shanxi, 710061, China

**Keywords:** gene signature, radio-sensitivity, radiotherapy, survival prediction, sarcoma

## Abstract

Adjuvant radiotherapy is an important clinical treatment option for the majority of sarcomas. The motivation of current study is to identify a gene signature and to predict radiosensitive patients who are most likely to benefit from radiotherapy. Using the public available data of soft tissue sarcoma from The Cancer Genome Atlas, we developed a cross-validation procedure for identifying a gene signature and predicting radiosensitive patients through. The result showed that the predicted radiosensitive patients who received radiotherapy had a significantly better survival with a reduced rate of new tumor event and disease progression. Strata analysis showed that the predicted radiosensitive patients had significantly better survival under radiotherapy independent of histologic types. A hierarchical cluster analysis was used to validate the gene signature, and the results showed the predicted sensitivity for each patient well matched the results from cluster analysis. Together, we demonstrate a radiosensitive molecular signature that can be potentially used for identifying radiosensitive patients with sarcoma.

## INTRODUCTION

Soft tissue sarcomas are rare and aggressive malignancies that develop from the mesenchymal tissue. The incidence rate has increased over the past 35 years as there are approximately 12,000 new cases of soft tissue sarcoma diagnosed and 4,800 deaths each year in the United States [1–3]. A recent report from the National Cancer Institute shows that the 5-year relative survival rate for soft tissue sarcoma is approximately 65% (http://www.cancer.gov/research/progress/snapshots/sarcoma) [4]. Systemic therapy options for sarcoma remain limited. Adjuvant radiotherapy plays a critical role in integrated multimodality treatment of sarcoma [5].

However, due to the complex heterogeneity of sarcoma, not all treated patients benefit from radiotherapy. For certain radiosensitive histological subtypes, such as myxoid liposarcoma, pre-operative radiotherapy may be particularly advantageous [6, 7]. Ewing's sarcoma is also considered as a relative radiosensitive type [8]. A retrospective study has shown that preoperative radiotherapy might be not suitable for all patients with primary soft tissue sarcoma of the limbs [9]. Late and chronic toxicities of radiotherapy, such as severe induration, loss of subcutaneous tissue, subcutaneous fibrosis are often concerned [5, 10, 11]. Radiation-induced bone fractures are serious complications occurring in 2-20% of patients treated with limb-sparing surgery and radiotherapy [12]. Therefore, the survival benefit of radiotherapy on soft tissue sarcomas have not been observed significantly [13–15]. We argue that if we can develop a radiosensitivity signature, we might be able to identify right patients for radiotherapy.

In the era of precision medicine, personalized radiation therapy, through the use of biomarkers to guide exclusive radiotherapy and/or combination therapy, has started to emerge in recent years [16]. Various gene signatures with specificity in terms of diagnosis, prognosis or prediction of a therapeutic response have been developed and validated [17, 18]. Gene signatures have been used to predict radiosensitive patients in many cancer types, including glioblastoma, cervical, breast, colorectal, head and neck cancer cells [19–23]. However, there is no effective radiosensitive gene signature well developed for sarcoma.

In this paper, we utilized the RNAseq data for soft tissue sarcoma from The Cancer Genome Atlas (TCGA, http://cancergenome.nih.gov/) to develop a radiosensitive gene signature for predicting radiosensitive patients. Since sarcoma is a rare disease, it is difficult to find another independent dataset with survival outcome and enough RNAseq data to do independent-sample validation. Furthermore, due to the limitation of the small sample size in the TCGA dataset, it is not ideal to do split-sample validation. To overcome these difficulties, we performed an internal cross validation via the cross-validated adaptive signature design that combined the gene signature development and the validation test in a single pivotal trial, as introduced by Freidlin and Simon (2005) and Freidlin (2010) [24, 25]. Following this novel idea, we extended this approach to the proportional hazard model and developed a radiosensitive gene signature for predicting radiosensitive patients in sarcoma.

## RESULTS

### Survival analysis on clinical information

Table 1 summarizes the results of the clinical information. Univariate and multivariable analyses showed that most of the clinical factors, including radiotherapy, are not significant predictors for overall survival. The poor associations between clinical factors and overall survival might suggest that genetic factors play an important role in predicting survival outcome of soft tissue sarcoma.

**Table 1:** Patients clinical characters and results of univariate and multivariate Cox regression analysis

Characteristic		No	Univariate analysis		Multivariable analysis	
			HR (95% CI)	*p* values	HR (95% CI)	*p* values
Gender						
	Female	138	1.00		1.00	
	Male	115	0.869(0.581-1.301)	0.496	0.843(0.517-1.374)	0.493
Age(median: 61, range: 20 to 90)						
	<60	112	1.00		1.00	
	≥60	141	1.322(0.879-1.988)	0.180	1.373(0.852-2.215)	0.193
Race						
	White	221	1.00		1.00	
	Nonwhite	24	1.406(0.675-2.929)	0.362	1.999(0.867-4.607)	0.103
	[Unknown]	8				
History of malignancy						
	No	213	1.00		1.00	
	Yes	40	0.941(0.534-1.659)	0.834	1.119(0.600-2.090)	0.723
Histologic diagnosis						
	LMS	100	1.00		1.00	
	DLS	58	1.195(0.725-1.972)	0.485	0.863(0.447-1.664)	0.659
	MFS+DT	25+2	0.769(0.373-1.585)	0.476	1.008(0.431-2.360)	0.985
	MPNST	9	1.040(0.322-3.367)	0.947	0.756(0.182-3.145)	0.700
	SS	10	0.978(0.350-2.735)	0.967	1.848(0.605-5.642)	0.281
	UPS	49	1.076(0.601-1.926)	0.806	1.138(0.563-2.299)	0.718
Margin status						
	Negative	134	1.00		1.00	
	Positive	71	1.780(1.105-2.866)	0.019	1.027(0.356-2.964)	0.957
	[Unknown]	48				
Residual tumor						
	R0	152	1.00		1.00	
	R1	66	2.140(1.365-3.355)	0.001	2.345(0.934-5.886)	0.068
	R2	8	12.601(5.579-28.459)	<.0001	10.430(3.266-33.304)	<.0001
	RX	27	2.165(1.140-4.109)	0.018	2.311(1.095-4.879)	0.028
Tumor depth						
	Superficial	20	1.00		1.00	
	Deep	184	2.529(0.692-9.247)	0.156	1.800(0.429-7.547)	0.408
	[Unknown]	49				
Tumor necrosis						
	0%	66	1.00		1.00	
	<10%	38	1.348(0.740-2.454)	0.328	1.629(0.717-3.701)	0.233
	≥10% ∼ 50%	61	1.484(0.853-2.582)	0.159	1.364(0.722-2.575)	0.332
	>50%	12	1.614(0.631-4.129)	0.314	1.515(0.474-4.846)	0.472
	[Unknown]	76				
Multifocal						
	NO	192	1.00		1.00	
	YES	39	2.178(1.351-3.512)	0.001	1.541(0.845-2.808)	0.158
	[Unknown]	22				
Radiotherapy						
	No	177	1.00		1.00	
	Yes	76	0.850(0.545-1.325)	0.473	0.855(0.488-1.498)	0.583
Chemotherapy						
	No	195	1.00		1.00	
	Yes	57	1.205(0.764-1.902)	0.423	1.216(0.696-2.127)	0.492
	[Unknown]	7				
Status						
	Dead	97				
	Censor	156				
Survival time (month)						
Median (95%CI): 65.4 (54.1-88.4)						
5-year survival rate (%): 54.80 (47.50-63.20)						

Note: nonwhite group including African American (18 patients) and Asian (6 patients); LMS: Leiomyosarcoma; DLS: Dedifferentiated liposarcoma; UPS: Undifferentiated Pleomorphic Sarcoma; MFS: Myxofibrosarcoma; DT: Desmoid Tumor; SS: Synovial Sarcoma; MPNT: Malignant Peripheral Nerve Sheath Tumors.

### Development of a radiosensitive gene signature

**Figure 1:**
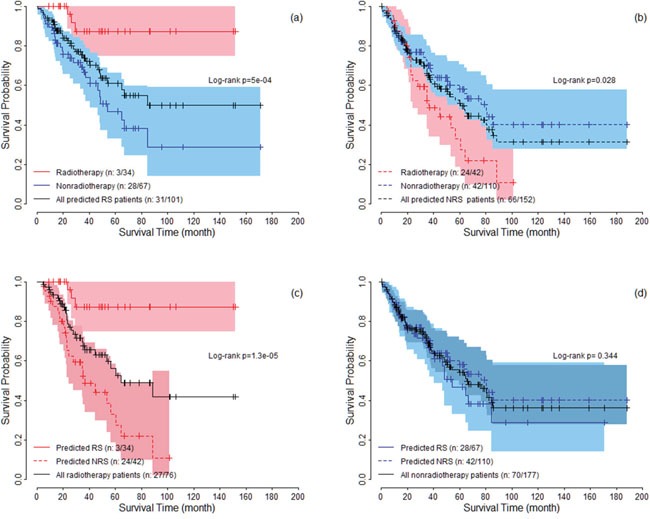
The survival curves under radiotherapy and nonradiotherapy for both predicted radiosensitive (RS) and nonradiosensitive (NRS) patients The colored areas denote the 95% confidence intervals for survival rate.

Following the proposed procedure, we analyzed the current data set to obtain the tuning parameters by 10-fold cross-validation. The 10 loops could produce 10 combinations of tuning parameters and the gene signature might be different. Theoretically, the reselection of the significant genes for different loops of the cross-validation is essential to the validity of the approach [26]. However, it does not mean that the classifications and selection are unstable or that the classifier will not predict accurately for independent data. Genomic signatures are generally not unique [25, 27]. As suggested by Freidlin [25], to save computational time, the first cross-validation subset could be used to select the tuning parameter. The minimum *p* value (*p*=4.810E-04) was reached when the top 26 significant genes were included with a threshold nHR of 0.035 for predicting the sensitive patients. Supplementry Figure 1 presents the *p*-values profiles by log-rank tests between radiotherapy and nonradiotherapy groups when different tuning parameters combinations were used in the first loop of cross-validation procedure. Supplementry Table 1 shows the genes included in the radiosensitive gene signature and their interaction effects with radiotherapy.

### Validation of the radiosensitivity prediction

Following the standard validation procedure, we predicted 101 patients as radiosensitive patients, and divided patients into four subgroups: predicted radiosensitive patients who received radiotherapy, predicted radiosensitive patients who did not receive radiotherapy, predicted nonradiosensitive patients who received radiotherapy and predicted nonradiosensitive patients who did not receive radiotherapy. We compared the survival for these four subgroups. Figure 1(a) shows the survival curves for predicted radiosensitive patients. The significant difference suggested that the predicted radiosensitive patients strongly benefited from radiotherapy compared with non-radiotherapy. Figure 1(b) shows significant differences between nonradiosensitive patients under radiotherapy and non-radiotherapy, suggesting that radiotherapy not only did not benefit, but worsen the survival for nonradiosensitive patients. We further compared the survival among radiosensitive and nonradiosensitive patients all under radiotherapy treatment, shown in Figure 1(c). As expected, strong positive effect of radiotherapy on radiosensitive patients were observed. In addition, there was no significant difference in survival between radiosensitive and nonradiosensitive patients who did not receive radiotherapy treatmentas shown in Figure 1(d). Taken together, as expected, the radiosensitive gene signature provide powerful predictive values for both radiosensitive and nonradiosensitive in radiotherapy.

**Figure 2:**
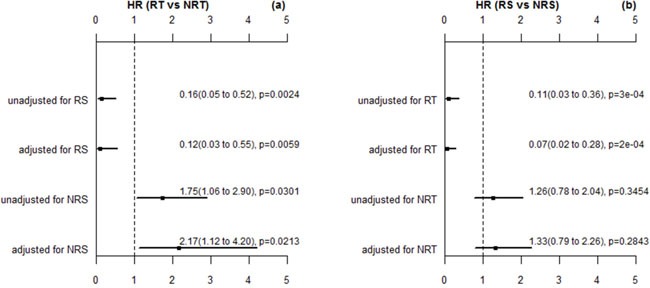
The HR estimation for radiotherapy (RT) verse nonradiotherapy (NRT) and radiosensitive (RS) verse nonradiosensitive (NRS) These p values here are estimated by wald test. The adjusted factors are gender, age, chemotherapy, histologic type, and residual rumor (the significant factor in multivariable analysis).

In addition, we further performed multivariable analysis using the Cox proportional hazard regression to assess the effect of radiotherapy on overall survival for radiosensitive and nonradiosensitive patients. The adjusted factors included age, gender, chemotherapy, historic type, and residual tumor (residual tumor is the only significant factor associated with overall survival in Table 1). Figure 2(a) shows that radiotherapy strongly improved the survival for radiosensitive patients, while for nonradiosensitive patients, radiotherapy might be a risk factor, with the adjusted HR as 2.17(1.12 to 4.2). When both radiosensitive and nonradiosensitive patients all received radiotherapy, radiosensitive patients had a significantly higher probability of survival than nonradiosensitive patients, while there is no significant difference in the probability of survival between radiosensitive and nonradiosensitive patients who did not receive radiotherapy, as shown in Figure 2(b). These results suggest that the prediction on radiosensitive patients were accurate and effective.

### Associations among radiotherapy and clinical assessments after adjuvant treatments

**Figure 3:**
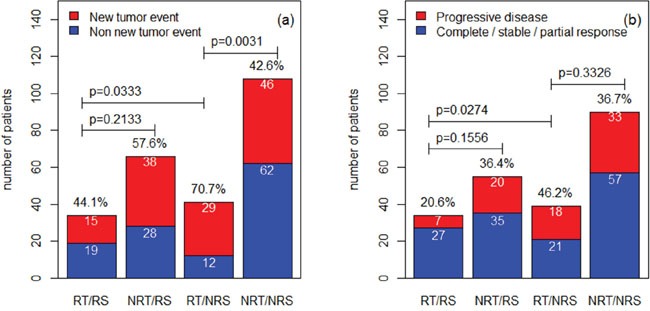
The comparisons among different groups for the rate of new tumor event and progressive disease The rates for different groups are compared by Fisher exact test. RT: radiotherapy; NRT: nonradiotherapy; RS: radiosensitive; NRS: nonradiosensitive.

To further validate the signature, we further compared the rate of new tumor event and progressive disease for the predicted radiosensitive and nonradiosensitive patients. According to TCGA, new tumor event is defined as metastatic, recurrent, and new primary tumor after initial treatment. Treatment response measures success of outcome at the completion of additional treatment. In current data, progressive disease group includes 78 patients. Non-progressive group includes complete response (127 patients), partial response (4 patients), and stable disease (9 patients). The results were summarized in Figure 3(a) for new tumor event and in Figure 3(b) for progressive disease. The results suggest that the predicted radiosensitive patients who received radiotherapy have a significant lower rate of new tumor event and disease progression. These results are consistent with the results derived from the above survival analysis, and further validated our prediction.

### Associations between radiosensitivity and clinical factors

**Figure 4:**
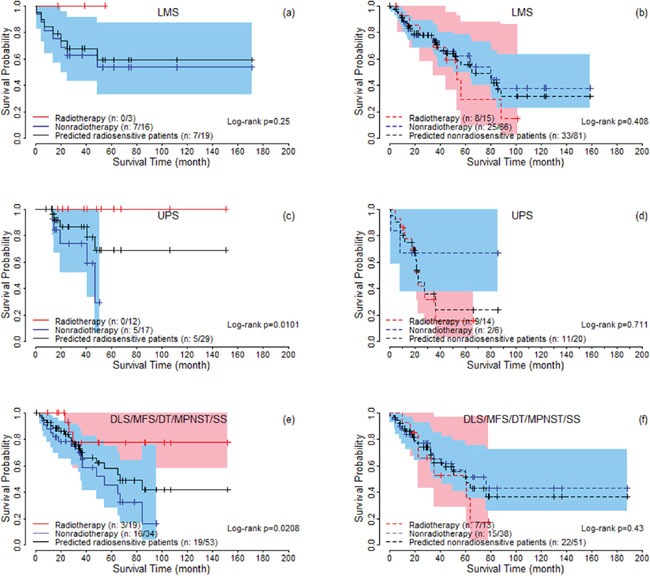
The survival curves under radiotherapy and nonradiotherapy for predicted radiosensitive and nonradiosensitive patients with different histologic types For DLS, MFS, MPNST, and SS, the proportion of predicted radiosensitive and nonradiosensitive are very similar and the sample sizes are also small for these groups. Therefore, they are combined together for logrank test (Figure 4e and 4f).

For the predicted radiosensitive and nonradiosensitive patients, we performed an analysis to test the association between predicted sensitivity and clinical factors, by both univariate and multivariable analysis. Supplementry Table 2 summarizes the results. Univariate and multivariable analysis both suggested that only histologic type significantly associates with predicted radiosensitivity. We performed strata analysis under LMS, UPS and other histologic types, respectively. Log-rank tests suggested that the predicted radiosensitive patients in the radiotherapy group had better survival compared with non-radiotherapy group, no matter which histologic types they were (Figure 4(4a, 4c, 4e)). For nonradiosensitive patients, radiotherapy might be a potential risk factor, although log-rank tests did not suggest a significant difference (Figure 4(4b, 4d, 4f)).

In addition, for patients who received radiotherapy, we compared the total dose for predicted sensitive (n=28) and nonsensitive patients (n=39) with available records. The medians of total dose (interquartile range) are 6300(6000-6420) and 6300 (6000-7010) for the two groups. There is no significant difference between the two groups with p value 0.1933 by Wilcoxon test. Moreover, there are 30 and 33 patients who received radiotherapy on primary tumor field for predicted radiosensitive and nonradiosensitive groups, respectively. There is no significant different between the distribution of radiation therapy site. The radiotherapy type is all external radiotherapy. These results suggest that total dose, radiation therapy site, and radiotherapy type are not a confound factor on the sensitivity prediction.

### Gene signature and cluster analysis

**Figure 5:**
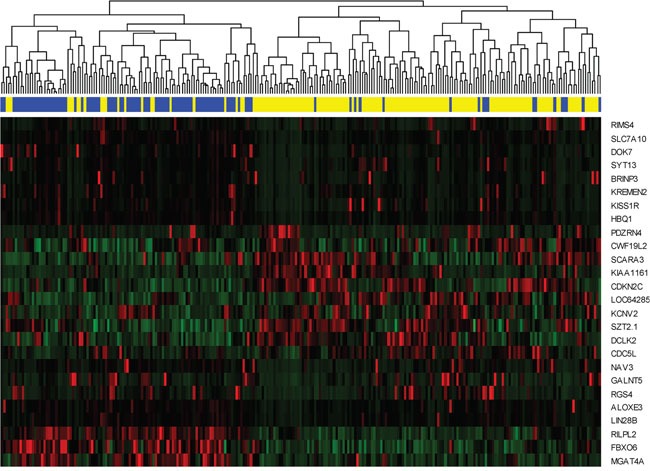
Hierarchical clustering analysis Hierarchical clustering was used to determine the expression pattern of selected 26 genes. The top blue and yellow bands denote the predicted radiosensitive and nonradiosensitive patients, respectively. Totally, 83 out of 101 predicted radiosensitive patients are classed at the left branch, and 126 out of 152 predicted nonradiosensitive patients are classed at the right branch.

We further extracted the expression pattern of the selected 26 genes to perform hierarchical clustering analysis by using R packages pheatmap. The results are presented in Figure 5. All of the patients were classified into two groups according to a hierarchical cluster analysis. The blue and yellow bar denoted the predicted radiosensitive and nonradiosensitive patients, respective. We can see that the predicted radiosensitive and nonradiosensitive patients were well matched with the result of hierarchical cluster based on the selected gene signature. More than 82% predicated radiosensitive and nonradiosensitive patients located on the left and right branch of the dendrogram, respectively. These results further validated our previous prediction.

## DISCUSSION

Incorporating radiotherapy with novel radiosensitive biomarkers or gene signatures might potentially increase the survival of patients with sarcoma. Although molecular mechanistic studies have shed lights for exploring radiosensitive gene signatures [28, 29], till now, knowledge is still limited about the molecular determinants of tumor radiatiosensitivity in the clinical setting. The inability to understand the fundamental molecular basis for sarcoma sensitivity or resistance to radiation prevents a risk-based clinical trial as well as target driven therapeutic strategies. In addition, due to the large number of genes available for analysis, the development of a reliable diagnostic classifier using early nonrandomized phase II data is often not feasible. Furthermore, validation data might also be limited for the rare sarcoma.

In this article, we followed and extended an adaptive cross-validated procedure to identify sarcoma patients that would be more sensitive to radiotherapy. The whole genome expression data was evaluated by testing the proposed model and new index (nHR). The radiosensitive gene signature including 26 significant genes was found to be predictive of radiosensitive patients. Since, no external validation data exists at this time, we verified our gene signature and sensitivity prediction from the following five aspects: (1) A 10-fold cross validation procedure (a standard validation procedure) to verify the predicted radiosensitive patients. The main results showed that the predicted radiosensitive patients who received radiotherapy had significantly better survival than both the radiosensitive patients without radiotherapy and nonradiosensitive patients who received radiotherapy (Figure 1). (2) After adjusted other clinical factors, multivariate analysis suggested that radiotherapy on the predicted radiosensitive patients was an independent benefit factor (Figure 2). (3) The reduced rate of new tumor event and progressive disease were observed for predicted radiosensitive patients who received radiotherapy, which further provided strong positive evidence for our prediction (Figure 3). (4) Although the histologic type was the only clinical factor strongly associated the predicted radiosensitivity, the survivals of the predicted radiosensitive patients who received radiotherapy were significantly better than radiosensitive patients without radiotherapy, no matter which histologic type they were (Figure 4). (5) The overlap of results from cluster analysis and predicted radiosensitive and nonradiosensitive patients also validated the radiosensitive gene signature (Figure 5). Taken together, these validation results reveal that the identified radiosensitive gene signature is a powerful biomarker for predicting which sarcoma patients would benefit from radiotherapy. Furthermore, the proposed model and cross-validation procedures are an effective approach for developing gene signatures and predicting sensitive patients for cancers beyond sarcoma.

The developed gene signature is easy to apply for predicting new patients. According to the estimation of each genes in gene signature, one just calculate the HR for each gene using the standardized expression value of RNAseq, then compare the product of these HR (nHR) with the threshod 0.035. The patients can be predicted as radiosensitive patients if their nHR less than the threshold.

Our analysis not only developed a radiosensitive gene signature, but also detected genes which may be potentially associated with the molecular basis of sarcoma. For example, LIN28B, might be a potential oncogenic driver for sarcoma. A previous report suggested that LIN28B was involved in a predictive network for osteosarcoma [30]. Emerging evidence indicates that LIN28B is an oncogenic driver in cancer stem cells [31]. LIN28B has been identified to be overexpressed in a wide range of solid tumors and hematological malignancies, such as pancreatic cancer, ovarian cancer, atypical teratoid/rhabdoid tumor, neuroblastoma, oral cancer, et al. [32–38] It might suggest that LIN28B could be a therapeutic target in sarcoma.

KISS1R, which is also on the gene signature list, is a predictive marker for pancreatic cancer, lung cancer, breast cancer, renal cell carcinomas. Patients with KISS1R expression compared to that without expression usually have a favorable prognosis. Targeting the KISS1R signaling axis is considered as a promising strategy to inhibit invasiveness and metastasis [39–44]. However, the possible association among KISS1R and sarcoma has not been reported. RGS4 and SLC7A10 are two important genes directly associated with sarcoma in previous reports [45–47]. Other genes, such as ALOXE3, HBQ1, KREMEN2, SYT13, DOK7, CDC5L, and FBXO6, have been reported in several cancers [48–53]. These results may provide helpful clues for further research in sarcoma.

## MATERIALS AND METHODS

### Study samples

All data including clinical information and normalized RNAseq expression were downloaded from The Cancer Genome Atlas (TCGA, http://cancergenome.nih.gov/) (update at March 2016). Clinical data is available for 261 patients. Expression data included 259 patients for 20502 genes with clear gene names after removing duplicated patients from raw data with 265 samples. Genes having a maximum expression value of 10 were excluded as they showed almost no expression. Genes with proportion of zero expression more than 75% were also removed. We then standardized the expression data and combined the overall survival and other information together. Next, we merged standardized expression data with clinical information, and removed the patients with missing radiotherapy information. This resulted in 253 patients with 18166 gene expression profiles for the final analysis. Finally, any missing values in clinical data were filled by multiple imputation using the R package mice. The cleaned clinical data are summarized in Table 1.

### Methods

#### Gene signature development

In the present study, the radiosensitive patients are defined as a group of patients who have higher probability of survival if they receive radiotherapy. To develop the patient radioactive sensitive signature for predicting radiosensitive patients, we used the following modeling assumption: there is a subset of *S* predictive (“sensitive”) genes that significantly interact with radiotherapy. The survival benefit of radiotherapy is associated with these predictive genes through the Cox proportional hazards model:

$$h(t|X)=h_{0}(t)\exp(r\lambda +x_{1}b_{1}+x_{2}b_{2}+\cdots +x_{S}b_{S}+rx_{1}i_{1}+rx_{2}i_{2}+\cdots +rx_{S}i_{S})$$

where *h*_0_(*t*) is the baseline hazard function; *λ* is the effect of radiotherapy; *r* is an indicator for radiotherapy with 1 indicating radiotherapy and 0 otherwise; *b*_1_ to *b*_S_ are the main effects for these *S* sensitive genes; *i*_1_ to *i*_S_ are radiotherapy-expression interaction effects that reflect the degree by which the effect of radiotherapy on survival is influenced by the expression levels of sensitive genes.

If the radiotherapy-expression interaction effects are negative, patients who overexpress the sensitive genes will have a higher survival probability under radiotherapy compared with non-radiotherapy. We assume that a fraction of the patient population overexpresses some (but not necessarily all) of the sensitive genes. The total Hazard Ratio (HR) would tend to be less than a preset threshold value (such as less than 0.5). Then, these patients who had a relative high probability of survival are called radiosensitive patients.

#### Cross-validation procedure

Freidlin and Simon (2005) and Freidlin (2010) developed a novel cross-validated adaptive signature design to identify sensitive patients in clinical trial for binary outcome [24, 25]. Following their framework, we extended and modified this approach to proportional hazards model and applied it to develop radiosensitive gene signature for current sarcoma data. A *K*-fold cross-validated procedure for gene signature development is described by the following three-step procedure.

Step 1: *Training step*. Split the data into *K* parts with the same sample size randomly (usually *K* = 10). Then, (*K* – 1) parts are used as training data to fit models and predict the radiosensitive patients in the left-out part (validation data). In the training data, for each gene *j*, fit a Cox proportional hazards model: $h(t|X)=h_{0}(t)\exp(r\lambda +x_{j}b_{j}+rx_{j}i_{j})$. Then, the p values for i_j_ were used to rank the genes.

Step 2: *Prediction step*. Use the top significant *g* genes to build a gene signature, and calculate an index, called nominal HR (nHR) by $\exp(r\lambda +\sum_{i}^{g}\left(x_{j}b_{j}+rx_{j}i_{j}\right))$, for patients in the validation data (*k*-th part). Here, *λ* could be the value averaged over the estimates from *g* single gene models. Patients in the validation set who has nHR lower than a specified threshold *R* will be classified as radiosensitive patients.

Step 3 *Validation step*. Cycling through the above two procedures, and validating on each of the *K* pieces in turn. Each study patient only appears once in one of the validation data. After the cross validation, each patient is classified as either radiosensitive or not. For radiosensitive patients, Log-rank tests are then performed to test the survival difference between radiotherapy and non-radiotherapy groups at a specified significant level, such as 0.05. A significant test result will indicate radiotherapy is beneficial for radiosensitive patients, then the gene signature is considered effective, and the prediction of radiosensitive patients is accurate.

In the above procedure, there are two key tuning parameters: *g* and *R* in the *Prediction step*. The optimal values of the tuning parameters *g* and *R* are usually not known in advance. Therefore, all the possible combinations for *g* and *R* could be tried and tested. One can use a nested inner loop of *K*-fold cross-validation approach on the training data to select the best tuning parameters values without affecting statistical validity of the procedure. An example of such procedure is provided in Supplementary Appendix.

In the above procedure, the 10-fold cross validation is recommended which permits the maximization of the portion of study patients contributing to the development of the diagnostic signature and the minimization of prediction error [54]. Beyond 10-fold cross validation, split sample method and leave-one-out cross-validation (LOOCV) are often mentioned in international validation. As known that split sample method usually provided poor performance on prediction, especially for small sample data. LOOCV could provide similar and stable results, compared with10-fold cross validation. However, LOOCV can be very time consuming to implement [54].

## SUPPLEMENTARY MATERIALS FIGURES AND TABLES






