# Supplementary material for: Development of a radiosensitivity gene signature for patients with soft tissue sarcoma
Source: Oncotarget. 2017 Mar 15;8(16):27428–39. doi: 10.18632/oncotarget.16194 (PMC5432346; doi:10.18632/oncotarget.16194)
Supplement: Supplementary file 1 [file oncotarget-08-27428-s001.pdf]

Development of a radiosensitivity gene signature for patients with soft tissue sarcoma

SUPPLEMENTARY APPENDIX, FIGURE AND TABLES

Appendix I: selection of tuning parameters *g* and *R*

In the *Training step*, including (*K*-1) parts patients, the tuning parameters (*g*, *R*) were selected empirically by choosing the values that gave the highest power to predict sensitive patients on a set of possible (*g*, *R*) combinations. In practice, we recommend the following approach based on 10-fold cross-validation to select the best combination from a set of M possible combinations (using *Training step* (*K*-1) patients only):

Part 1: Split the data to *T* parts with the same sample size randomly, here *T*=10 is recommended. Remove the *t*-th part patients and carry out step 1 of the three-step procedure (described in Materials and Methods) on the remaining patients. Then, using step 2 of the three-step procedure, determine if the *t*-th part patients is classified as sensitive according to different possible tuning parameter combinations. Empirically, we try top *g* from 1 to 200 significant genes (with significant radiotherapy-expression interactions), and set *R* that ranges from 0.005 to 0.5 by 0.005. Then, a total M=20000 possible tuning parameter combinations would be tried.

Part 2: Repeat part 1 only from *t*=1 to 10, let each study patient to be predicted exactly one time under a tuning parameter combinations. Try all M=20000 possible tuning parameter combinations and form M subsets of sensitive patients, each corresponding to a set of tuning parameters.

Part 3: For each of the M subsets, compare survival among the predicted sensitive patients who received the radiotherapy and the predicted sensitive patients who did not received the radiotherapy. Select the tuning parameter combination that provides the smallest P value in Logrank test. This tuning parameter combination would be used to filter the sensitive patients on validation patients at Step 2: *Prediction step*.

This approach preserves the validity of predicting radiosensitive patients in *k*-th subset, as only the data from the (*K*-1) parts is used to determine the tuning parameters. This procedure is a nested inner loop of *K*-fold cross-validation applied only in *Training step* (*K*-1) patients. In this procedure, *T*=10 is recommended, 10-fold cross validation usually have small and stable bias and error [1]. One may try leaving-one-out cross-validation (LOOCV) to get a stable result. However, LOOCV can be very time consuming to implement.

In addition, for different (*K*-1) patients in *Training step*, the tuning parameters (*g*, *R*) might be different. Theoretically, the reselection of the tuning parameters (*g*, *R*) or significant genes for different loops of the cross-validation is essential to the validity of the approach [2]. However, it does not mean that the classifications and selection are unstable or that the classifier will not predict accurately for independent data. Good genomic signatures are generally not unique. [3, 4]. As described by Freidlin [4], to save computational time, the first cross-validation subset could be used to select the turning parameter (*g*, *R*).

REFERENCES

1. Molinaro AM, Simon R, Pfeiffer RM. Prediction error estimation: a comparison of resampling methods. Bioinformatics. 2005; 21:3301-3307.
2. Simon R, Radmacher MD, Dobbin K, McShane LM. Pitfalls in the Use of DNA Microarray Data for Diagnostic and Prognostic Classification. Journal of the National Cancer Institute. 2003; 95:14-18.
3. Fan C, Oh DS, Wessels L, Weigelt B, Nuyten DSA, Nobel AB, van't Veer LJ, Perou CM. Concordance among Gene-Expression–Based Predictors for Breast Cancer. New England Journal of Medicine. 2006; 355:560-569.
4. Freidlin B, Jiang W, Simon R. The cross-validated adaptive signature design. Clinical cancer research. 2010; 16:691-698.

Appendix II: a work flow to show the whole procedure of the proposed method

(1) Split the data to 10 parts, use the blue parts (training data set) to train the data, and obtain the tuning parameter. According to the tuning parameter, select the sensitive patients (red part) from the test data (green part). Cycle the above procedure, like from 1th to 10th part. Then, compare the survival among radiothearpy and nonradiotherapy gourp. A significant difference suggests the sensitivity prediction is good.

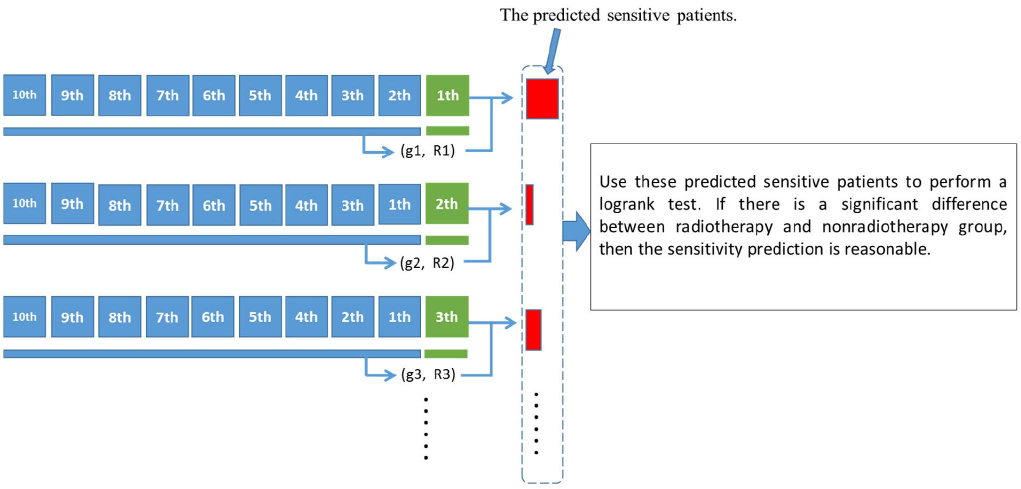

In the above procedure (1), the 10 loops could produce 10 combinations of tuning parameters and the gene signature might be different. To save computational time, the first cross-validation subset could be used to select the tuning parameter.

(2) the tuning parameters (g, R) for different loop are different in the above procedure. Here, we take (g1, R1) as an example to show How to get the tuning parameters.

First, split the blue part (training data set) to T=10 parts. Then, try possible tuning parameter combinations (g', R') to predict "sensitive patients" (orange part) from test data (black part). Perform a logrank test on these "sensitive patients". Select the tuning parameter combination (g', R') that provides the smallest P value in Logrank test. Then this tuning parameter combination (g', R') can be used as (g1, R1) in above procedure.

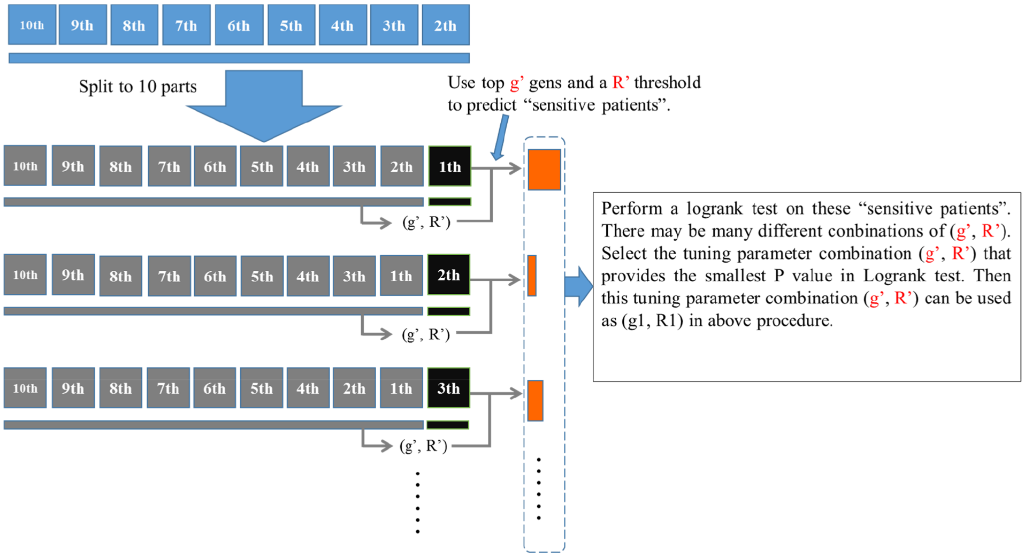

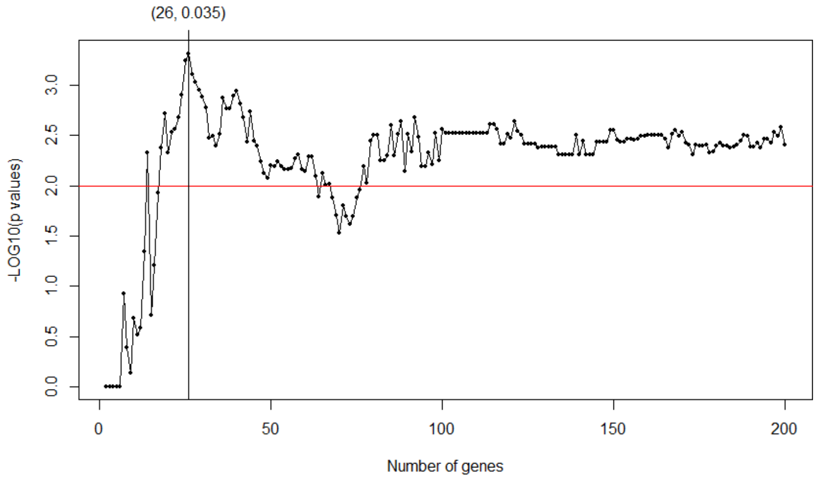

Supplementary Figure 1: The –log10(*p*-values) profile by Logrank tests between radiotherapy and nonradiotherapy groups for predicted radiosensitive patients. It can be found that gene signatures including top 26 significant genes with a threshold nHR=0.035 can provide a powerful prediction with the smallest *p* value (p=4.810E-04).

Supplementary Table 1: The 26 genes included in the radiosensitive gene signature and their interaction effects with radiotherapy

See Supplementary File 1

Supplementary Table 2: Association analysis among predicted radiosensitivity and clinical factors

See Supplementary File 2
