# Supplementary material for: Development of a radiosensitivity gene signature for patients with soft tissue sarcoma
Source: Oncotarget. 2017 Mar 15;8(16):27428–39. doi: 10.18632/oncotarget.16194 (PMC5432346; doi:10.18632/oncotarget.16194)
Supplement: Supplementary file 2 [file oncotarget-08-27428-s002.docx]

**Table S1 The 26 genes included in the radiosensitive gene signature and their interaction effects with radiotherapy.**

|  | Gene names | Main effects  of genes (SE) | p values | Main effects of  radiotherapy(SE) | p values | Interaction  effects(SE) | p values |
| --- | --- | --- | --- | --- | --- | --- | --- |
| 1 | LIN28B | 1.804(0.317) | 1.272E-08 | -0.304(0.229) | 0.186 | -1.642(0.321) | 3.096E-07 |
| 2 | KISS1R | 1.008(0.189) | 1.026E-07 | -0.255(0.227) | 0.263 | -1.076(0.234) | 4.172E-06 |
| 3 | ALOXE3 | 2.049(0.464) | 1.001E-05 | -0.342(0.230) | 0.137 | -1.954(0.468) | 2.952E-05 |
| 4 | GALNT5 | -0.169(0.134) | 0.207 | 0.031(0.228) | 0.890 | 1.472(0.371) | 7.273E-05 |
| 5 | DCLK2 | -0.183(0.130) | 0.159 | -0.088(0.229) | 0.699 | 0.815(0.206) | 7.686E-05 |
| 6 | SLC7A10 | 1.421(0.314) | 6.057E-06 | -0.280(0.230) | 0.222 | -1.252(0.318) | 8.238E-05 |
| 7 | HBQ1 | 2.123(0.521) | 4.623E-05 | -0.333(0.230) | 0.148 | -2.053(0.526) | 9.545E-05 |
| 8 | KCNV2 | -0.020(0.121) | 0.870 | -0.205(0.238) | 0.389 | 0.850(0.224) | 1.487E-04 |
| 9 | RGS4 | 0.017(0.098) | 0.858 | -0.062(0.228) | 0.784 | 0.917(0.245) | 1.829E-04 |
| 10 | KREMEN2 | 0.871(0.202) | 1.546E-05 | -0.239(0.230) | 0.297 | -0.812(0.220) | 2.194E-04 |
| 11 | NAV3 | -0.072(0.135) | 0.595 | 0.073(0.228) | 0.749 | 1.966(0.533) | 2.240E-04 |
| 12 | BRINP3 | -0.085(0.116) | 0.462 | -0.141(0.229) | 0.540 | 0.784(0.212) | 2.240E-04 |
| 13 | CDKN2C | -0.034(0.111) | 0.757 | -0.126(0.233) | 0.589 | 0.966(0.265) | 2.668E-04 |
| 14 | MGAT4A | 0.177(0.102) | 0.083 | -0.599(0.321) | 0.062 | -1.765(0.485) | 2.722E-04 |
| 15 | FBXO6 | 0.159(0.153) | 0.300 | -0.583(0.315) | 0.064 | -1.420(0.394) | 3.139E-04 |
| 16 | SYT13 | 0.881(0.182) | 1.39E-06 | -0.233(0.230) | 0.311 | -0.701(0.195) | 3.319E-04 |
| 17 | KIAA1161 | -0.301(0.164) | 0.066 | -0.157(0.230) | 0.494 | 0.798(0.223) | 3.503E-04 |
| 18 | LOC642852 | 0.045(0.128) | 0.727 | -0.283(0.244) | 0.245 | 0.678(0.190) | 3.544E-04 |
| 19 | RIMS4 | 0.073(0.066) | 0.270 | -0.151(0.230) | 0.512 | 0.640(0.181) | 4.213E-04 |
| 20 | RILPL2 | 0.117(0.107) | 0.274 | -0.340(0.260) | 0.190 | -1.526(0.436) | 4.686E-04 |
| 21 | SZT2.1 | -0.132(0.116) | 0.254 | -0.242(0.240) | 0.312 | 0.710(0.203) | 4.864E-04 |
| 22 | CWF19L2 | -0.210(0.143) | 0.142 | -0.171(0.236) | 0.467 | 1.008(0.290) | 5.026E-04 |
| 23 | SCARA3 | -0.269(0.126) | 0.032 | -0.157(0.229) | 0.492 | 0.787(0.228) | 5.526E-04 |
| 24 | CDC5L | 0.016(0.093) | 0.865 | -0.246(0.238) | 0.300 | 0.742(0.215) | 5.657E-04 |
| 25 | DOK7 | 0.638(0.164) | 9.95E-05 | -0.191(0.227) | 0.399 | -0.714(0.209) | 6.344E-04 |
| 26 | PDZRN4 | -0.438(0.195) | 0.025 | -0.105(0.230) | 0.648 | 1.093(0.321) | 6.706E-04 |
