# Supplementary material for: Development of a radiosensitivity gene signature for patients with soft tissue sarcoma
Source: Oncotarget. 2017 Mar 15;8(16):27428–39. doi: 10.18632/oncotarget.16194 (PMC5432346; doi:10.18632/oncotarget.16194)
Supplement: Supplementary file 3 [file oncotarget-08-27428-s003.docx]

**Table S2** **Association study among predicted radiosensitivity and clinical factors**.

| Characteristic |  | Sensitive patients | Nonsensitive patients | Univariate analysis | Multivariate analysis* |
| --- | --- | --- | --- | --- | --- |
|  |  |  |  | *p* values | *p* values |
| Gender |  |  |  | 0.1495 | 0.6454 |
|  | Female | 49 | 89 |  |  |
|  | Male | 52 | 63 |  |  |
| Age(median: 61, range: 20 to 90) | | | | 0.0624 | 0.5301 |
|  | <60 | 37 | 75 |  |  |
|  | ≥60 | 64 | 77 |  |  |
| race |  |  |  | 0.965 | 0.7427 |
|  | White | 89 | 132 |  |  |
|  | NonWhite | 9 | 15 |  |  |
| History of malignancy | |  |  | 0.3730 | 0.8895 |
|  | No | 82 | 131 |  |  |
|  | Yes | 19 | 21 |  |  |
| Histologic diagnosis* | |  |  | 6.775e-06 |  |
|  | LMS | 19 | 81 |  |  |
|  | DLS | 30 | 28 |  | 0.0016 |
|  | MFS+DT | 13 | 14 |  | 0.0868 |
|  | MPNST | 4 | 5 |  | 0.8106 |
|  | SS | 6 | 4 |  | 0.0441 |
|  | UPS | 29 | 20 |  | 0.0032 |
| Margin status | |  |  | 1.00 | 0.4086 |
|  | Negative | 52 | 82 |  |  |
|  | Positive | 27 | 44 |  |  |
| Residual tumor | |  |  | 0.8974 |  |
|  | R0 | 61 | 91 |  |  |
|  | R1 | 27 | 39 |  | 0.8773 |
|  | R2 | 2 | 6 |  | 0.2598 |
|  | RX | 11 | 16 |  | 0.9744 |
| Tumor depth | |  |  | 1.00 | 0.6113 |
|  | Superficial | 8 | 12 |  |  |
|  | Deep | 76 | 108 |  |  |
| Tumor necrosis | |  |  | 0.8677 |  |
|  | 0% | 29 | 37 |  |  |
|  | <10% | 14 | 24 |  | 0.9692 |
|  | ≥10% ~ 50% | 23 | 38 |  | 0.8532 |
|  | >50% | 5 | 7 |  | 0.4566 |
| Multifocal | |  |  | 0.4425 | 0.2447 |
|  | NO | 73 | 119 |  |  |
|  | YES | 18 | 21 |  |  |
| Chemotherapy |  |  |  | 0.0125 | 0.1361 |
|  | NO | 86 | 109 |  |  |
|  | YES | 14 | 43 |  |  |
| Tumor site |  |  |  | 0.0050 |  |
|  | Abdominal | 39 | 71 |  |  |
|  | CNH* | 9 | 9 |  | 0.2110 |
|  | Extremity | 40 | 44 |  | 0.1013 |
|  | Gynecological | 4 | 24 |  | 0.7355 |
|  | Superficial | 8 | 4 |  | 0.0623 |

Note: LMS: Leiomyosarcoma; DLS: Dedifferentiated liposarcoma; UPS: Undifferentiated Pleomorphic Sarcoma; MFS: Myxofibrosarcoma; DT: Desmoid Tumor; SS: Synovial Sarcoma; MPNT: Malignant Peripheral Nerve Sheath Tumors. CNH: Chest (13 patients), Head and Neck (5 patients). Extremity: including upper and lower Extremity. The missing data were impute by multiple imputation procedure in R packages “mice” when multivariate analysis is implement.
